# Effects of a cardiac rehabilitation program on anthropometric and functional parameters: an observational pre-post study

**DOI:** 10.1186/s43044-026-00752-5

**Published:** 2026-05-22

**Authors:** Estrella García-Sánchez, Mirian Santamaría-Peláez, Jerónimo J. González-Bernal, Josefa González-Santos, Jessica Fernández-Solana, Rodrigo Vélez-Santamaría

**Affiliations:** 1 Hospital Universitario de Burgos, Burgos, Spain; 2 University of Burgos, Burgos, Spain

**Keywords:** Cardiac rehabilitation, Physical activity, Ergoesphirometry, Metabolic equivalent of task, NYHA functional classification

## Abstract

**Introduction:**

Cardiac rehabilitation is a multidisciplinary intervention designed to improve functional capacity and quality of life in patients with cardiovascular diseases. However, its impact on anthropometric and functional parameters remains an important area of study. This study evaluates the effects of a cardiac rehabilitation program on body mass index, abdominal circumference, blood pressure, heart rate, energy expenditure measured in METs, and NYHA functional classification.

**Methods:**

An observational pre-post study without a control group was conducted with 287 patients diagnosed with cardiovascular disease who completed a cardiac rehabilitation program. Anthropometric and functional variables were measured before and after the intervention. Statistical analysis included paired-sample t-tests and the Wilcoxon test for NYHA classification, with a significance level of *p* < 0.05.

**Results:**

Significant reductions were observed in BMI (pre: 27.79 ± 4.31 kg/m²; post: 27.11 ± 4.09 kg/m²; *p* < 0.001) and abdominal circumference (pre: 100.38 ± 11.16 cm; post: 97.33 ± 12.28 cm; *p* < 0.001). Systolic and diastolic blood pressure significantly decreased (*p* < 0.001). An increase in energy expenditure measured in METs was found (pre: 7.89 ± 2.68; post: 10.52 ± 2.63; *p* < 0.001), as well as in maximum heart rate. Additionally, NYHA functional classification improved (Z = -9.356, *p* < 0.001) with a reduction in the proportion of patients in classes III and II and an increase in class I.

**Conclusion:**

The cardiac rehabilitation program resulted in significant improvements in body composition, blood pressure, functional capacity, and NYHA classification. These findings support the importance of cardiac rehabilitation as an effective strategy in managing patients with cardiovascular diseases. Further strategies should be implemented to improve adherence and assess the long-term impact of the intervention.

## Introduction

Cardiac rehabilitation (CR) is a structured programme designed to improve recovery and quality of life in patients who have suffered cardiac events, such as myocardial infarction or heart failure [1, 2]. These programmes combine supervised exercise, education about healthy habits and counselling to reduce the risk of future cardiovascular problems and optimise health outcomes [3, 4].

The effectiveness of CR has been widely demonstrated, prompting its integration into post-cardiac event care. Several studies have shown that CR not only significantly decreases the risk of recurrent cardiovascular events but also improves health parameters such as body mass index (BMI), blood pressure and functional capacity [3, 5]. In particular, body composition, as measured by BMI and abdominal girth, has been a key focus of CR research. Participants in these programmes have been shown to experience improvements in these parameters, which is associated with a reduction in cardiovascular risk factors such as hypertension and elevated cholesterol levels [6, 7].

In addition, CR emphasises the importance of physical exercise and nutritional counselling, components that work synergistically to facilitate weight management and improve overall cardiovascular health [8]. This multidimensional approach allows for personalised interventions tailored to individual patient needs, further reinforcing the relevance of CR in clinical practice.

One of the most significant benefits of CR is the improvement in blood pressure, especially in patients with treatment-resistant hypertension. Structured lifestyle modifications, including changes in diet and regular physical activity, have shown significant reductions in blood pressure levels, contributing to better long-term cardiovascular outcomes [9, 10].

Furthermore, the New York Heart Association (NYHA) functional classification plays a key role in CR, as it allows healthcare professionals to assess and adapt rehabilitation strategies based on patients’ functional limitations and symptoms. This facilitates personalisation of treatment with the aim of improving exercise tolerance and overall well-being [1, 2, 4].

Recent evidence has also highlighted the effectiveness of home-based and remotely supervised cardiac rehabilitation programs, particularly through the use of wearable sensors and telemonitoring systems. These approaches have demonstrated positive effects on functional capacity, exercise adherence, and cardiovascular risk management, while improving accessibility for patients with geographical or mobility limitations [11, 12].

Despite its benefits, CR has generated some debate, especially in relation to its impact on weight loss and the so-called ‘obesity paradox’. Some studies suggest that, although CR may lead to modest weight reduction, the relationship between weight change and cardiovascular outcomes is complex and requires further investigation [7, 13]. Furthermore, the need to improve patient adherence to these programmes highlights the importance of understanding barriers to participation, as greater engagement is crucial to maximise health benefits and prevent future cardiac events [5, 14].

In this context, the present study aims to evaluate the effects of a cardiac rehabilitation program (CRP) on anthropometric and functional parameters through a pre-post observational design in patients with cardiovascular pathology. Key indicators such as BMI, blood pressure, abdominal circumference, ergometry performance, and NYHA functional classification will be analyzed before and after the intervention. Functional capacity assessment through METs and exercise-based performance tests remains a key component in cardiac rehabilitation research and clinical practice, as these measures provide important prognostic information related to cardiovascular morbidity, hospitalization, and mortality risk [15].

## Methods

### Participants

This study included 287 patients with a diagnosis of cardiac pathology, who were referred by the cardiologist responsible for the Cardiac Rehabilitation Unit to assess their eligibility for the outpatient physical training programme. After the assessment, those who met the inclusion criteria completed the rehabilitation programme.

To participate in the study, patients had to have a confirmed diagnosis of cardiovascular disease (CVD), have been referred to the Cardiac Rehabilitation Unit by the Cardiology Service, sign the informed consent form prepared specifically for this research and complete the CRP. Patients were excluded if they had any medical condition that prevented them from performing the prescribed physical exercise, those who refused to participate or those who did not complete the programme.

This study was approved by the Medicines Research Ethics Committee of the Burgos and Soria Health Area (Ref. CEIm 2569–22 June 2021) and followed the guidelines established in the Declaration of Helsinki of the World Medical Association.

### Procedure

This study followed an observational pre-post design with a single experimental group consisting of patients with cardiovascular pathology. The sample size was not calculated, and a control group was not included for ethical considerations. As the treatment aimed to enhance participants’ well-being and provide direct health benefits, it was deemed unethical to withhold the intervention from any group. Therefore, rather than including a conventional control group, a study design was selected in which all participants received the treatment, ensuring fairness and equal benefit for everyone involved.

**Fig. 1:**
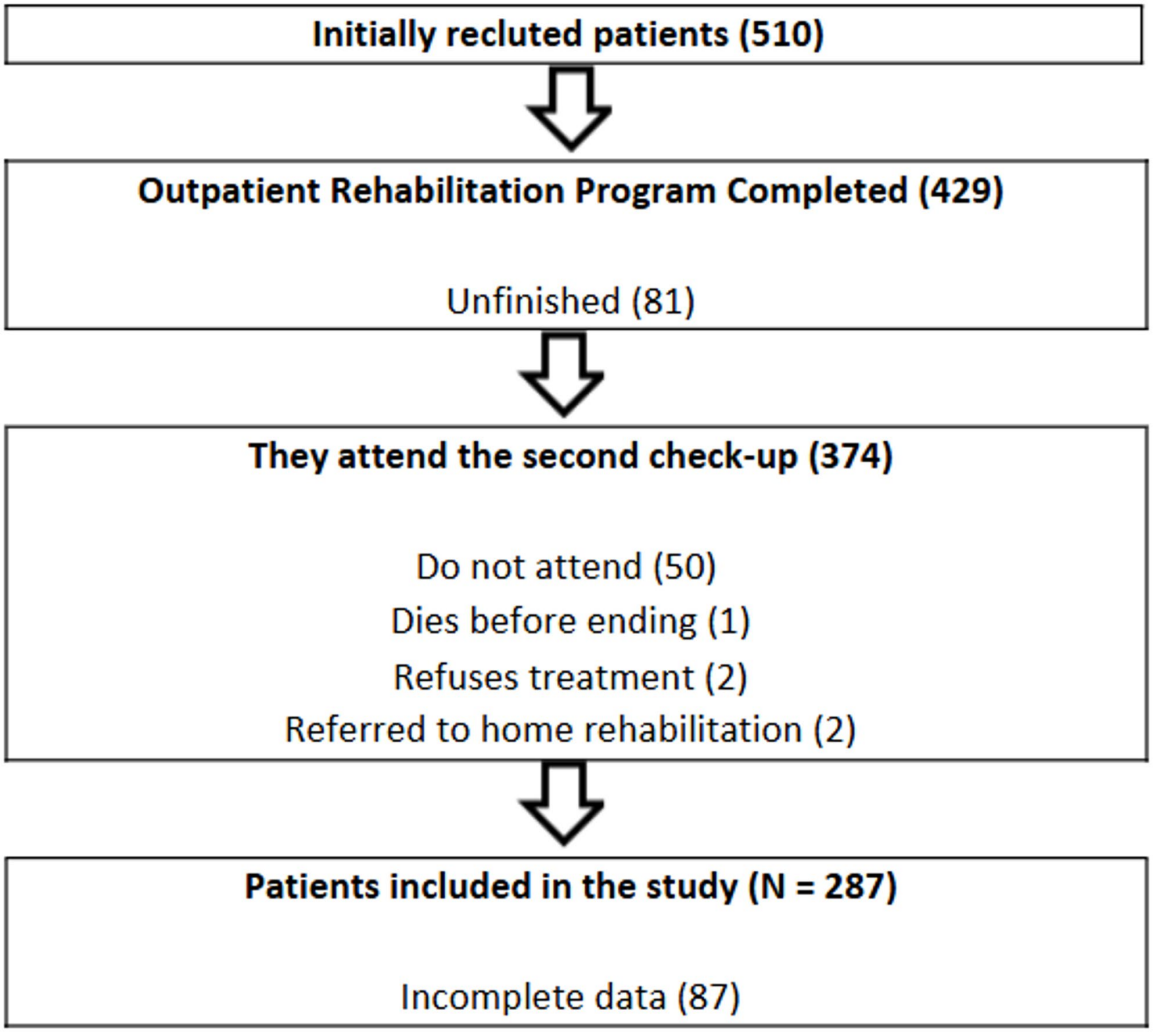
Flow chart

After verifying the inclusion criteria, data collection was performed at the Rehabilitation Service of the University Hospital of Burgos during the initial consultation (pretest) of patients eligible to participate in the CRP. A physician specialised in physical medicine and rehabilitation was in charge of the collection of clinical data, extracted from the patients’ medical history, sociodemographic data and assessment scales were also recorded at the consultation. This study flow chart is shown in Fig. 1.

After referrals from the cardiology department, the patients attended the Cardiac Rehabilitation Unit of the University Hospital of Burgos. During the initial consultation, a cardiologist conducted a comprehensive clinical evaluation, which included a medical history review and an assessment of complementary tests such as the lipid profile, electrocardiogram, and echocardiography. If a recent stress test was unavailable, either an exercise test or cardiopulmonary exercise testing was performed. After that, cardiovascular risk factors, dietary habits, smoking history and social environment were assessed in the nursing consultation, and health education and documentation explaining the programme were provided. Next, a rehabilitation physician reviewed the medical history, confirmed adherence to pharmacological treatment, and conducted a general physical examination to assess the patient’s eligibility for participation in the CRP. Finally, a physiotherapist from the Cardiac Rehabilitation Unit provided another explanation of the program to the patients and oversaw its implementation.

The programme duration was between one and two months, consisting of training sessions two to three times a week. This duration was established according to the standard clinical practice and organizational protocols of the Cardiac Rehabilitation Unit of the University Hospital of Burgos, taking into account patient availability, clinical stability, and healthcare resource management. Each session was structured in four phases. The first phase consisted of a ten-minute warm-up, in which exercises were performed to mobilise large muscle groups by means of flexions, extensions, lateralisations and rotations at the cervical, upper and lower limb and trunk levels. Each exercise was repeated approximately five times.

In the second phase, aerobic exercise was performed for 35 min, using cycloergometers or treadmills. The intensity was adjusted according to the maximal heart rate (HRF) obtained in the exercise test, using Karvonen’s formula [16, 17]. During the first third of the sessions, work was performed at 50% of the MHRF, increasing to 60% in the second third and 80% in the last phase [18–20]. In patients assessed by ergospirometry, the intensity was set based on the heart rate obtained between the first and second ventilatory threshold [18, 21]. In general, training was initiated with a continuous regime, aiming to maintain a stable heart rate at a sustained intensity. Subsequently, an interval training regime was introduced based on the results of the Conconi test [22].

The third phase centered on strength training, performed twice a week. The large muscle groups worked with weights of between one and two kilograms, performing four to five repetitions per exercise. Special emphasis was placed on correct technical execution and controlled breathing, ensuring exhalation during effort and inspiration during the relaxation phase.

Finally, the fourth phase included stretching and flexibility exercises, lasting between five and ten minutes. Static stretching and balance exercises were performed with the aim of improving flexibility and favouring the return to calm after training.

Since psychological well-being plays a key role in CR, the programme incorporated psychological therapy sessions, both group and individual, according to the patient’s needs. These sessions were conducted by a clinical psychologist. Additionally, a physiotherapist conducted relaxation workshops lasting between twenty and thirty minutes, focusing on diaphragmatic breathing techniques and progressive muscle relaxation. One-hour educational talks were also held each week, covering topics such as heart-healthy nutrition, knowledge of CVD, associated pharmacology, physical activity and sexuality, cardiovascular risk factors and smoking cessation strategies.

Before each session, patients’ vital signs, such as blood pressure and heart rate, were recorded. Additionally, abdominal circumference and body weight were measured on a weekly basis. Patients who required further support were referred to the Smoking Cessation Unit, Clinical Psychology, or Mental Health services, depending on their specific needs.

Three months after completing the CRP (post-test), a new round of data collection was conducted. The gathered information was then entered into a database for statistical analysis. During the same consultation, patients were reminded of the importance of sustaining the lifestyle changes achieved throughout the program.

### Instruments

In this study, different measurement instruments were used to assess changes in anthropometric and functional parameters of participants before and after intervention in the CRP.

Body weight (kg) and body mass index (BMI, kg/m²) were recorded using a digital scale with a calibrated measuring rod before each measurement. BMI was calculated using the standard formula: weight in kilograms divided by height in metres squared (kg/m²), following the World Health Organization (WHO) recommendations for classification of nutritional status [23].

Abdominal circumference (cm) was measured with a flexible tape measure, placed at the midpoint between the lower edge of the last rib and the iliac crest, in a standing position and with the patient in normal expiration, according to the guidelines of the International Society for the Advancement of Kinanthropometry (ISAK) [24]. This parameter is essential for cardiovascular risk assessment, as abdominal fat accumulation is associated with an increased risk of cardiometabolic disease [25].

Systolic and diastolic blood pressure (mmHg) was measured with a validated digital sphygmomanometer following the recommendations of the European Society of Cardiology (ESC) and the European Society of Hypertension (ESH) [26]. Three measurements were taken with an interval of one minute between each, using the average of the last two measurements to minimise variability.

Resting heart rate (bpm) and maximum heart rate (bpm) were recorded using a heart rate monitor during the exercise assessment. The maximum heart rate was estimated using the exercise test and, failing that, the Tanaka et al. formula (208 − 0.7 × age) to estimate the patient’s cardiovascular capacity [27].

Energy expenditure was calculated from the data obtained in the maximal exercise test, expressing functional capacity in units of Metabolic Equivalent of Task (METs). This metric is considered a key indicator of cardiorespiratory fitness and functional capacity in patients with CVD, according to the American College of Sports Medicine (ACSM) guidelines [28].

Finally, the New York Heart Association (NYHA) functional classification was used to assess the impact of rehabilitation on patients’ functional capacity. This system categorises individuals into four classes, from no functional limitations in Class I to inability to perform any physical activity in Class IV [29]. The NYHA classification is a validated tool widely used in clinical practice to assess heart failure progression and response to treatment [30].

All measurements were performed by trained professionals using standardised protocols to ensure accuracy and reliability of the data.

### Statistical analysis

All statistical analyses were conducted using IBM SPSS Statistics (Version 25.0). A descriptive analysis was performed for all variables, calculating means and standard deviations for quantitative variables and frequencies and percentages for categorical variables. A significance threshold of *p* < 0.05 was established for all analyses.

To compare pre- and post-intervention measurements for quantitative variables, the paired samples t-test was used. Effect sizes were estimated using Cohen’s d, with values between 0.2 and 0.3 considered small, between 0.3 and 0.8 considered moderate, and above 0.8 considered large [31].

For the NYHA functional classification, given its ordinal nature, the Wilcoxon signed-rank test was applied to compare pre- and post-intervention measurements. Effect sizes were calculated using r for Wilcoxon, with values between 0.1 and 0.3 considered small, between 0.3 and 0.5 considered moderate, and above 0.5 considered large [32, 33].

## Results

The study included a total of 287 participants, with a mean age of 60.90 ± 10.75 years (range: 15–84). The gender distribution was predominantly male (85.7% men, 14.3% women). Regarding place of residence, 74.6% of the participants lived in Burgos, while 12.9% lived within 60 km and 12.5% lived more than 60 km away.

In Table 1, the means and standard deviations of the quantitative variables in the pre- and post-intervention measurements are presented.

**Table 1:** Descriptive statistics of pre- and post- variables

Variable	Pre (Mean ± SD)	Post (Mean ± SD)
Weight (Kg)	78.19 ± 14.97	76.68 ± 13.86
BMI (Kg/m2)	27.79 ± 4.31	27.11 ± 4.09
Abdominal perimeter (cm)	100.38 ± 11.16	97.33 ± 12.28
Systolic blood pressure (mmHg)	132.27 ± 15.72	125.10 ± 14.70
Diastolic blood pressure (mmHg)	78.23 ± 7.52	74.45 ± 10.64
Heart rate (bpm)	67.23 ± 12.96	65.82 ± 10.92
Maximum heart rate (bpm)	122.89 ± 19.51	127.27 ± 19.84
Energy expenditure (METs)	7.89 ± 2.68	10.52 ± 2.63

SD: Standard deviation; BMI: Body Mass Index; MET: Metabolic Equivalent of Task

Table 2 shows the functional classification (NYHA) of the participants before and after the intervention.

**Table 2:** Descriptive statistics: NYHA pre and post

NYHA classification	Pre (frequency)	Pre (%)	Post (frequency)	Post (%)
I	170	59.2%	251	87.5%
II	76	26.5%	32	11.1%
III	41	14.3%	4	1.4%

Paired samples t-tests were conducted to evaluate pre- and post-intervention differences in the quantitative variables. The results are presented in the following Table 3. The results indicate significant improvements in all variables, with effect sizes ranging from small to large. Notably, the increase in energy expenditure measured in METs shows a large effect size (d = -1.74), reflecting a substantial improvement in participants’ functional capacity.

**Table 3:** T-test results for paired samples

Variable	Mean difference post – pre (95% CI)	t-value	*p*-value
Weight (kg)	-1.51 (-2.19, -0.84)	-4.41	< 0.001
BMI (kg/m2)	-0.68 (-0.83, -0.54)	-9.42	< 0.001
Abdominal perimeter (cm)	-3.05 (-3.83, -2.28)	-7.77	< 0.001
Systolic blood pressure (mmHg)	-7.18 (-9.24, -5.11)	-6.84	< 0.001
Diastolic blood pressure (mmHg)	-3.78 (-5.04, -2.53)	-5.93	< 0.001
Heart rate (bpm)	-1.41 (-2.73, -0.08)	-2.09	0.037
Maximum heart rate (bpm)	4.38 (2.07, 6.70)	3.72	< 0.001
Energy expenditure (METs)	2.64 (2.46, 2.82)	29.43	< 0.001

CI: Confidence Interval; BMI: Body Mass Index; MET: Metabolic Equivalent of Task

In Table 3, some values show negative differences in post - pre weight, BMI, abdominal circumference, blood pressure and heart rate that indicate a reduction in these parameters, which is a positive result in terms of cardiovascular health. In contrast, the increase in METs consumption and maximal heart rate suggests better functional capacity, which is interpreted as a favorable effect of rehabilitation.

The Wilcoxon signed-rank test was performed to assess changes in NYHA functional classification pre- and post-intervention. The results are presented in Table 4. The results indicate a significant improvement in functional classification after the intervention (Z = -9.356, *p* < 0.001), with a large effect size (*r* = -0.55), suggesting a substantial improvement in patients’ functional capacity.

**Table 4:** Wilcoxon signed-rank test for NYHA functional classification

Variable	*N*	Test STATISTIC	Z	*p*-value	*r* (effect size)
FC_PRE - FC_POST	287	194.000	-9.356	< 0.001	− 0.55

FC: Functional Classification (NYHA)

## Discussion

Participants in the CR programme showed significant reductions in body mass index (BMI) and abdominal girth. In addition, an improvement in functional capacity was evident, reflected in the increase in metabolic equivalents (METs) achieved during exercise testing. Improvements in blood pressure and resting heart rate were also observed. Finally, the New York Heart Association (NYHA) functional classification showed a favourable evolution, with a significant proportion of patients transitioning to lower functional classes.

CR has a significant impact on various health parameters, including the Body Mass Index (BMI), which is a critical measure of body composition and overall health. The program is designed to be a comprehensive, multidisciplinary approach tailored to the individual needs of patients with cardiovascular disease (CVD) [6].

Recent updates to CR protocols emphasize the importance of body composition assessments beyond traditional metrics such as weight and body mass index (BMI) [6, 9]. Measuring factors like waist circumference and waist-to-hip ratio can provide a clearer picture of a patient’s health and associated cardiovascular risks [7]. Improvements in body composition, particularly reductions in abdominal obesity, have been linked to lowered cardiovascular risk, which underscores the relevance of BMI in the context of CR [7]. Similar findings have been reported in previous clinical studies showing improvements in functional capacity and waist-to-hip ratio following participation in cardiac rehabilitation programs in patients with coronary artery disease [34].

The reduction in BMI and abdominal girth observed in this study is consistent with previous research reporting decreases in these parameters following participation in CR programmes. For example, a study conducted in Spain found significant improvements in patients’ weight and abdominal circumference after a CR programme [35]. The decrease in central adiposity is particularly relevant, as abdominal obesity is associated with an increased risk of adverse cardiovascular events [36].

CRP have shown varying effects on weight management among participants. While the primary aim of these programs is to improve cardiovascular health, weight loss can also be a beneficial outcome. Research indicates that participation in CR typically results in modest weight loss, averaging around 3% to 4% of body weight [13, 37]. However, it is important to note that the degree of weight loss may not be significant enough to alter long-term cardiovascular outcomes.

The increase in METs achieved during exercise testing suggests an improvement in the cardiorespiratory fitness of patients. This finding is supported by studies indicating that functional capacity measured in METs is one of the best prognostic markers in patients with ischaemic heart disease (ICHD). In addition, improved functional capacity translates into greater independence in daily activities and improved quality of life. Improvements in functional capacity measured through METs have also been associated with reduced cardiovascular mortality and hospitalization risk in patients with cardiovascular disease [4, 7]. Similarly, favorable changes in NYHA functional classification are considered clinically relevant prognostic indicators linked to lower symptom burden and better long-term cardiovascular outcomes [30].

CRPs have been shown to have a significant impact on blood pressure, particularly for individuals with treatment-resistant hypertension. Research indicates that a structured diet and exercise program, such as the Dietary Approaches to Stop Hypertension (DASH) eating plan, can lead to substantial reductions in blood pressure among participants [3, 5]. In a study involving 140 adults with resistant hypertension, those who engaged in a supervised lifestyle modification program demonstrated a 7-point reduction in systolic blood pressure over a typical day, while a self-guided group showed no change in their blood pressure levels [5]. The American Heart Association recommends physical activity as an optimal first-line treatment for adults with elevated blood pressure and low heart disease risk. This recommendation underscores the importance of lifestyle interventions in managing hypertension effectively. Additionally, the improvements observed in participants of the structured program extend beyond just blood pressure; they also exhibited enhanced aerobic fitness and better overall heart health indicators, which suggest a lowered risk of future cardiovascular events [5]. Furthermore, the physiological responses to lifestyle modifications highlight how stress can influence blood pressure. Chronic stress and anxiety have been linked to elevated blood pressure levels due to the body’s fight-or-flight response, which releases adrenaline and increases heart rate. Managing stress through lifestyle interventions is crucial in reducing the risk of developing or exacerbating CVD over time [2, 5]. It is important to note that the observed improvements cannot be attributed exclusively to physical exercise, since the rehabilitation program also incorporated nutritional education, psychological support, smoking cessation strategies, and pharmacological monitoring, all of which may have contributed to the clinical outcomes observed.

Although improvements in blood pressure and resting heart rate were observed, it is important to note that some patients did not reach the established therapeutic targets. This finding highlights the need for additional or more intensive interventions in certain patient subgroups to achieve optimal control of these parameters. Previous studies have shown that CR can improve blood pressure and heart rate control, but the response may vary according to individual patient characteristics [38].

The favorable evolution in NYHA functional classification observed in this study indicates a reduction in symptomatology and improved exercise tolerance. This result is consistent with research showing improvements in functional class in patients with heart failure after participation in CRP [39]. Improvement in NYHA classification is associated with improved quality of life and a reduction in hospitalisations for cardiac decompensation.

The NYHA classification is essential for tailoring CR interventions to meet individual patient needs. By understanding a patient’s functional classification, healthcare providers can design appropriate exercise regimens and other therapeutic strategies aimed at improving exercise tolerance, reducing symptoms, and enhancing overall quality of life. Regular assessments using the NYHA system during rehabilitation allow for adjustments in the treatment plan to optimize patient outcomes [1, 2, 6]. Current international literature also emphasizes the importance of individualized and remotely supervised cardiac rehabilitation strategies tailored to patients’ functional capacity and clinical characteristics. These personalized approaches may further improve adherence, accessibility, and long-term functional outcomes [40].

Analysis of the NYHA functional classification shows a significant change, with an increase in the proportion of patients in Class I and a reduction in Classes II and III. This change is clinically relevant, as improvement in NYHA rating is associated with increased exercise tolerance, improved quality of life, and lower risk of hospitalization for heart failure. In particular, the reduction in Class III patients is significant, as these patients tend to have greater functional limitations and a worse prognosis [1, 2, 6]. These findings reinforce the positive impact of cardiac rehabilitation on functional recovery, although it would be advisable to evaluate its long-term sustainability through prolonged follow-up.

Although the results of the study show statistically significant differences in variables such as body mass index (BMI), blood pressure and abdominal circumference, it is important to analyze their clinical relevance. The reduction in systolic and diastolic blood pressure, although significant, should be interpreted in the context of cardiovascular risk reduction. Previous studies suggest that reductions of 5–10 mmHg in systolic blood pressure may be associated with a decreased risk of cardiovascular events [26]. Therefore, it is essential to complement these findings with longitudinal evaluations to determine the persistence of the changes and their impact on cardiovascular morbidity and mortality.

Although some anthropometric changes, such as reductions in body weight and BMI, were modest in magnitude despite reaching statistical significance, improvements in functional capacity measured through METs and changes in NYHA classification may be considered more clinically meaningful, given their known association with prognosis, exercise tolerance, and cardiovascular outcomes.

Despite the positive results, this study has some limitations. First, the absence of a control group limits the ability to establish causal relationships between the intervention and the observed improvements, although this decision was made for ethical reasons to ensure that all eligible patients had access to cardiac rehabilitation. Second, the short-term follow-up did not allow assessment of long-term adherence to lifestyle changes or the sustainability of the observed benefits over time. Although significant improvements were observed after the intervention, longer-duration cardiac rehabilitation programs may potentially achieve greater and more sustained benefits in functional capacity, cardiovascular risk reduction, and long-term adherence to healthy lifestyle habits. Finally, potential confounding factors such as medication adjustments, psychosocial influences, smoking status, comorbidity burden, and other concomitant interventions were not specifically controlled for in the analysis and may have influenced the outcomes. Future studies should consider alternative controlled designs, such as delayed intervention models, historical control groups, or propensity score matching approaches, as well as longer follow-up periods of at least 6 to 12 months to confirm these findings, evaluate the sustainability of benefits, and assess clinically relevant outcomes such as hospital readmissions and mortality, while minimizing internal validity risks such as regression to the mean or spontaneous recovery.

While the study assumes that improvements in anthropometric and functional parameters are due to cardiac rehabilitation, alternative hypotheses, such as spontaneous changes in patient health, placebo effects, or the influence of other concomitant treatments, have not been explored. Participation in a rehabilitation program could favor adherence to healthy habits independently of the structured intervention. To improve the validity of the results, future studies should include comparison groups or designs that control for variables such as self-efficacy and patient motivation, factors that may influence clinical outcome.

The study sample was predominantly male, with women being underrepresented. This limitation may affect the generalizability of the findings, as sex-related differences in participation, adherence, and response to cardiac rehabilitation programs have been previously described in the literature [41]. Future studies should aim for a more balanced representation of female participants to better evaluate potential sex-specific differences in rehabilitation outcomes.

The findings of this study support the effectiveness of CRP in improving anthropometric and functional parameters in patients with CVD. However, further efforts are required to ensure sustained adherence to recommendations and to maximise the long-term benefits of these interventions. Implementation of educational strategies and continuous follow-up may improve adherence and thus long-term outcomes [36].

## Conclusions

The results of this study confirm that CR is an effective strategy to improve anthropometric and functional parameters in patients with CVD. Reductions in body mass index and abdominal circumference were observed, as well as an improvement in functional capacity measured in METs.

In addition, CR contributed to a reduction in blood pressure and resting heart rate. An improvement in NYHA functional classification was also evident, reflecting increased exercise tolerance and improved quality of life.

Despite the positive results, the absence of a control group and the short-term follow-up are limitations to be considered.

In conclusion, these results underscore the value of structured cardiac rehabilitation and call for expanded access and sustained follow-up to optimize cardiovascular outcomes.

## Data Availability

The datasets presented in this article are not readily available because the the data are part of an ongoing study. Requests to access the datasets should be directed to mspelaez@ubu.es.

## References

[1] Ozemek C, Bonikowske A, Christle J, Gallo P (2025) ACSM’s Guidelines for Exercise Testing and Prescription. Lippincott Williams & Wilkins

[2] Rehabilitation AA (2013) of C& P. Guidelines for Cardia Rehabilitation and Secondary Prevention Programs-(with Web Resource). Human Kinetics

[3] Epstein E, Rosander A, Pazargadi A, Taub P (2020) Cardiac rehab for functional improvement. Curr Heart Fail Rep Springer 17:161–170 10.1007/s11897-020-00462-2 32514659

[4] Dibben G, Faulkner J, Oldridge N, Rees K, Thompson DR, Zwisler A-D et al (2021) Exercise-based cardiac rehabilitation for coronary heart disease. Cochrane Database of Systematic Reviews. John Wiley & Sons, Ltd; 10.1002/14651858.CD001800.pub4 PMC8571912 34741536

[5] Rashidi A, Whitehead L, Halton H, Munro L, Jones I, Newson L (2025) The changes in health-related quality of life after attending cardiac rehabilitation: A qualitative systematic review of the perspective of patients living with heart disease. PLoS One. Public Library of Science San Francisco, CA USA; ;20:e0313612 10.1371/journal.pone.0313612 PMC11781667 39883647

[6] Bäck M, Hansen TB, Frederix I (2017) Cardiac rehabilitation and exercise training recommendations, cardiac rehabilitation: Rationale, indications and core components. Retrieved June 9:2019

[7] Servey JT, Stephens M (2016) Cardiac rehabilitation: improving function and reducing risk. Am Fam Physician 94:37–43 27386722

[8] Brown TM Core Components Anchor Both Existing and Emerging Models of Cardiopulmonary Rehab

[9] Lewis CE, McTigue KM, Burke LE, Poirier P, Eckel RH, Howard BV et al (2009) Mortality, health outcomes, and body mass index in the overweight range: a science advisory from the American Heart Association, vol 119. Circulation. Lippincott Williams & Wilkins, pp 3263–3271 10.1161/CIRCULATIONAHA.109.192574 19506107

[10] Lavie CJ, Milani RV, Ventura HO (2009) Obesity and cardiovascular disease: risk factor, paradox, and impact of weight loss. J Am Coll Cardiol. American College of Cardiology Foundation Washington, DC; ;53:1925–32 10.1016/j.jacc.2008.12.068 19460605

[11] Antoniou V, Davos CH, Kapreli E, Batalik L, Panagiotakos DB, Pepera G (2022) Effectiveness of home-based cardiac rehabilitation, using wearable sensors, as a multicomponent, cutting-edge intervention: a systematic review and meta-analysis. J Clin Med MDPI 11:3772 10.3390/jcm11133772 PMC9267864 35807055

[12] Ramachandran HJ, Jiang Y, Tam WWS, Yeo TJ, Wang W (2022) Effectiveness of home-based cardiac telerehabilitation as an alternative to Phase 2 cardiac rehabilitation of coronary heart disease: a systematic review and meta-analysis. Eur J Prev Cardiol. Oxford University Press; ;29:1017–43 10.1093/eurjpc/zwab106 PMC8344786 34254118

[13] Gao C, Yue Y, Wu D, Zhang J, Zhu S (2025) Effects of high-intensity interval training versus moderate-intensity continuous training on cardiorespiratory and exercise capacity in patients with coronary artery disease: A systematic review and meta-analysis. PLoS ONE 20.Public Library of Science San Francisco, CA USAe0314134 10.1371/journal.pone.0314134 PMC11841918 39977401

[14] Aspry KE, Van Horn L, Carson JAS, Wylie-Rosett J, Kushner RF, Lichtenstein AH et al (2018) Medical nutrition education, training, and competencies to advance guideline-based diet counseling by physicians: a science advisory from the American Heart Association, vol 137. Circulation. Lippincott Williams & Wilkins Hagerstown, MD;, pp e821–e841 10.1161/CIR.0000000000000563 29712711

[15] Pepera G, Sandercock GRH (2022) Incremental shuttle walking test to assess functional capacity in cardiac rehabilitation: A narrative review. Int J Ther Rehabil MA Healthc Lond 29:1–10

[16] Karvonen J, Vuorimaa T (1988) Heart rate and exercise intensity during sports activities: practical application. Sports medicine, vol 5. Springer, pp 303–311 10.2165/00007256-198805050-00002 3387734

[17] Mj K (1957) The effects of training on heart rate: a longitudinal study. Ann med exp biol fenn 35:307–315 13470504

[18] Alemán JA, de Baranda Andujar PS, Ortín EJO (2014) Guía para la prescripción de ejercicio físico en pacientes con riesgo cardiovascular. Seh-Lelha

[19] Mezzani A, Hamm LF, Jones AM, McBride PE, Moholdt T, Stone JA et al (2013) Aerobic exercise intensity assessment and prescription in cardiac rehabilitation: a joint position statement of the European Association for Cardiovascular Prevention and Rehabilitation, the American Association of Cardiovascular and Pulmonary Rehabilitation and the Canadian Association of Cardiac Rehabilitation. Eur J Prev Cardiol. Sage Publications Sage UK: London, England; ;20:442–67 10.1177/2047487312460484 23104970

[20] Swain DP, Franklin BA (2006) Comparison of cardioprotective benefits of vigorous versus moderate intensity aerobic exercise. Am J Cardiol. Elsevier; ;97:141–7 10.1016/j.amjcard.2005.07.130 16377300

[21] Gaskill SE, Ruby BC, Walker AJ, Sanchez OA, SERFASS RC, LEON AS (2001) Validity and reliability of combining three methods to determine ventilatory threshold. Med Sci Sports Exerc LWW 33:1841–1848 10.1097/00005768-200111000-00007 11689733

[22] Conconi F, Ferrari M, Ziglio PG, Droghetti P, Codeca L (1982) Determination of the anaerobic threshold by a noninvasive field test in runners. J Appl Physiol 52:869–873 7085420 10.1152/jappl.1982.52.4.869

[23] Organization WH (2000) Obesity: preventing and managing the global epidemic: report of a WHO consultation. World Health Organization 11234459

[24] M-JM SA, Olds T, De Ridder H (2011) International Standards for Anthropometric Assessment. ISAK, Lower Hutt, New Zealand

[25] Després J-P (2012) Body fat distribution and risk of cardiovascular disease: an update. Circulation. Lippincott Williams & Wilkins Hagerstown, MD; ;126:1301–13 10.1161/CIRCULATIONAHA.111.067264 22949540

[26] Williams B, Mancia G, Spiering W, Agabiti Rosei E, Azizi M, Burnier M et al (2018) 2018 ESC/ESH Guidelines for the management of arterial hypertension: The Task Force for the management of arterial hypertension of the European Society of Cardiology (ESC) and the European Society of Hypertension (ESH), vol 39. Eur Heart J. Oxford University, pp 3021–3104 10.1093/eurheartj/ehy339 30165516

[27] Tanaka H, Monahan KD, Seals DR (2001) Age-predicted maximal heart rate revisited. J Am Coll Cardiol. American College of Cardiology Foundation Washington, DC; ;37:153–6 10.1016/s0735-1097(00)01054-8 11153730

[28] Bayles MP (2023) ACSM’s exercise testing and prescription. Lippincott williams & wilkins

[29] Committee NYHAssociationC (1979) Nomenclature and criteria for diagnosis of diseases of the heart and great vessels. Little, Brown Medical Division

[30] Yancy CW, Jessup M, Bozkurt B, Butler J, Casey DE Jr, Colvin MM et al (2017) 2017 ACC/AHA/HFSA focused update of the 2013 ACCF/AHA guideline for the management of heart failure: a report of the American College of Cardiology/American Heart Association Task Force on Clinical Practice Guidelines and the Heart Failure Society of America. J Am Coll Cardiol. American College of Cardiology Foundation Washington DC; ;70:776–803 10.1016/j.jacc.2017.04.025 28461007

[31] Cohen J (2013) Statistical power analysis for the behavioral sciences. routledge

[32] Rosenthal R (1984) Meta-analytic procedures for social science research Sage Publications: Beverly Hills, 148 pp. Educational Researcher. Sage publications Sage CA: Thousand Oaks, CA; 1986;15:18–20

[33] Fritz CO, Morris PE, Richler JJ (2012) Effect size estimates: current use, calculations, and interpretation. J Exp Psychol Gen Am Psychol Association 141:2 10.1037/a0024338 21823805

[34] Sahabazi Deh Sokhteh A, Pishkar Z, Rafizadeh O, Yaghoubinia F (2021) The effect of cardiac rehabilitation program on functional capacity and waist to hip ratio in patients with coronary artery disease: A clinical trial. Japan J Nurs Sci Wiley Online Libr 18:e12386 10.1111/jjns.12386 33107209

[35] Pujalte MF, Richart-Martínez M, Perpiñá-Galvañ J (2022) Análisis de la efectividad de la rehabilitación cardíaca en España: una revisión sistemática exploratoria. An Sist Sanit Navar. SciELO Espana

[36] Fernández Coronado R, Olórtegui Yzu A (2023) Efectividad de la prevención terciaria en la calidad de vida y control de los factores de riesgo en pacientes con cardiopatía coronaria isquémica. Archivos peruanos de cardiología y cirugía cardiovascular. 4:88–95 10.47487/apcyccv.v4i3.323 PMC10688413 38046229

[37] Lara-Breitinger K, Lynch M, Kopecky S (2021) Nutrition intervention in cardiac rehabilitation: a review of the literature and strategies for the future. J Cardiopulm Rehabil Prev LWW 41:383–388 10.1097/HCR.0000000000000660 34727557

[38] Castro-Conde A, Abeytua M, Esteban VIA, Pérez PC, González-Gallarza RD, Benito FG et al (2021) Factibilidad y resultados de un programa de rehabilitación cardiaca intensiva. Perspectiva del estudio aleatorizado MxM (Más por Menos). Rev Esp Cardiol. Elsevier; ;74:518–25 10.1016/j.rec.2020.03.029 32807709

[39] Álvarez-Martínez P, Alonso-Calvete A, Justo-Cousiño LA, González-González Y (2022) Eficacia de las diferentes modalidades de ejercicio terapéutico en rehabilitación cardiaca tras infarto de miocardio. Revisión de la literatura. An Sist Sanit Navar. SciELO Espana

[40] Pepera G, Antoniou V, Su JJ, Lin R, Batalik L (2024) Comprehensive and personalized approach is a critical area for developing remote cardiac rehabilitation programs. World J Clin Cases 12:2009 38680265 10.12998/wjcc.v12.i12.2009 PMC11045502

[41] Resurrección DM, Moreno-Peral P, Gomez-Herranz M, Rubio-Valera M, Pastor L, Caldas de Almeida JM et al (2019) Factors associated with non-participation in and dropout from cardiac rehabilitation programmes: a systematic review of prospective cohort studies. European Journal of Cardiovascular Nursing. Oxford University Press; ;18:38–47 10.1177/1474515118783157 29909641

